# Newspaper Cures

**Published:** 1870-07

**Authors:** 


### NEWSPAPER CURES.

QUITE a number of newspapers and periodicals have employed doctors to write for them. The more a physician knows, the less likely is he to have time to write for newspapers at a dollar a foot. Nine articles out of ten will detail the symptoms of the disease written about, and then proceed to give the cure by drugs, patent contrivances, or whiskey.

It is of the first importance, in all cases of sickness, to ascertain what is the matter. If every disease had but one symptom, which was peculiar to it, then it might be well to teach the public what symptoms meant; but so far from this being the case, almost every disease has a variety of symptoms, most of them symptoms in common with several other ailments. Hence only an educated and experienced physician can decide with reasonable certainty what any set of symptoms indicates; to commit an error here, and advise a remedy, may be the death of the patient. It is then most unwise for unprofessional persons to be studying symptoms, and to prescribe for them is actually dangerous.

Every man ought to have his family physician, just as every man has his lawyer, his tailor, or his cobbler. Don't be feeling about in the dark, and imagine this, that, and the other is the matter; relieve yourself of painful uncertainties and burdening responsibilities, by consulting your medical adviser, when you have any persistent troublesome sensation. For the multitude of little symptoms which now and then present themselves, the best plan is to eat a little less for a day or two, take some recreation in the country, or extra rest and sleep and warmth at home; and in multitudes of cases the symptoms will promptly abate and finally disappear altogether. But if, in spite of rest and warmth and quiet, the symptoms remain unchanged except for the worse, then promptly advise with your physician and take nothing except by his direction. If you do not attempt to mend your watch or even an old shoe, surely the body, so valuable to you and so delicately framed, should be put in skillful hands when repairs are needed.

